# Organ-specific redox and trace element modulation and hematological adaptation following hyperbaric oxygenation and physical activity in rats

**DOI:** 10.3389/fmed.2026.1820821

**Published:** 2026-06-29

**Authors:** Aleksandra Obradovic, Aleksandra Nenadovic, Nikola Mitovic, Rada Jeremic, Predrag Brkic, Teodora F. Vidonja Uzelac, Una Jovana Vujacic, Milan Ivanov, Sasa Jankovic, Andrija Vukovic, Silvio R. De Luka, Sanjin Kovacevic, Jelena Nesovic Ostojic

**Affiliations:** 1 Faculty of Sport and Physical Education, University of Belgrade, Belgrade, Serbia; 2 Department of Pathological Physiology, Faculty of Medicine, University of Belgrade, Belgrade, Serbia; 3 Department of Medical Physiology, Faculty of Medicine, University of Belgrade, Belgrade, Serbia; 4 Department of Physiology, Institute for Biological Research “Sinisa Stankovic”, National Institute of the Republic of Serbia, University of Belgrade, Belgrade, Serbia; 5 Department of Cardiovascular Physiology, Institute for Medical Research, National Institute of Republic of Serbia, University of Belgrade, Belgrade, Serbia; 6 Institute of Meat Hygiene and Technology, Belgrade, Serbia

**Keywords:** hyperbaric oxygen, oxidative stress, physical activity, macroelements, trace elements, Wistar rats

## Abstract

**Introduction:**

Oxidative stress represents an imbalance between the production of reactive oxygen species and the capacity of antioxidant defense systems to neutralize them. Both acute physical activity and hyperbaric oxygen exposure can influence redox homeostasis; however, their combined effects on systemic and tissue-specific oxidative status and trace element balance remain insufficiently understood. The aim of this study was to investigate the effects of hyperbaric oxygen preconditioning on oxidative stress parameters, antioxidant defense mechanisms, and elemental homeostasis following acute physical activity in previously untrained rats.

**Methods:**

Male Wistar rats were randomly assigned to four groups: control, physical activity (PA), hyperbaric oxygenation (HBO), and PA following HBO exposure (HBO + PA).

**Results:**

Across most measured parameters, no significant interaction between HBO and PA was detected, suggesting that their effects act largely independently. Instead, several significant main effects were identified. In particular, HBO increased erythrocyte count, hemoglobin concentration, and hematocrit, while erythrocyte indices remained unchanged, and were accompanied by increased total protein, albumin, and urea levels. HBO exerted a significant main effect on WBC count, while both HBO and PA were associated with lower lymphocyte counts. Systemic oxidative stress markers showed minimal alterations. An exception was catalase activity, which exhibited significant interaction. Nevertheless, distinct tissue-specific responses were observed. HBO was associated with increased lipid peroxidation in liver tissue, whereas in kidney tissue, HBO reduced lipid peroxidation and attenuated PA-associated depletion of sulfhydryl groups, highlighting organ-dependent redox modulation. Elemental findings further confirmed the predominance of main effects. In kidney tissue, HBO was linked to lower potassium, magnesium, phosphorus, zinc and lead levels, while PA was associated with lower iron and arsenic concentrations, and both interventions contributed to decreased sodium and mercury levels. In liver tissue, both PA and HBO were associated with reduced sodium levels, whereas HBO was associated with lower potassium and PA with higher magnesium concentrations. Phosphorus, manganese, copper, cobalt, and selenium demonstrated significant main effects; however, *post hoc* analyses did not reveal specific differences between individual experimental groups.

**Conclusion:**

Collectively, these findings suggest that short-term HBO and acute PA are associated with distinct organ-specific responses in redox balance and elemental homeostasis, predominantly through independent rather than interactive mechanisms.

## 1 Introduction

Oxidative stress is a physiological condition characterized by an imbalance between the production of reactive oxygen species (ROS) and the capability of antioxidant defense systems to neutralize them. ROS are continuously generated in cells as byproducts of normal metabolic processes, particularly during mitochondrial oxidative phosphorylation. In normal conditions, their levels are tightly regulated by enzymatic and non-enzymatic antioxidant mechanisms. However, when ROS production exceeds the capacity of cellular antioxidant defenses, their accumulation leads to oxidative stress and subsequent cellular damage (1, 2). Disturbances in redox homeostasis commonly affect lipid peroxidation, alter the activities of antioxidant enzymes, and change the levels of non-enzymatic antioxidants. Such alterations have long been recognized as important contributors to the pathogenesis of numerous pathological conditions, including cardiovascular, metabolic, and neurodegenerative diseases (2). At the same time, oxidative stress can also arise as a consequence of physiological stress, such as intense physical activity (PA).

During exercise, increased oxygen consumption and enhanced mitochondrial activity promote ROS production. Acute high-intensity aerobic or anaerobic exercise, particularly in previously untrained individuals, has been associated with elevated levels of oxidative stress markers (3, 4). The level of oxidative stress induced by exercise depends largely on the intensity and duration of the activity. However, regular PA also stimulates adaptive responses that enhance antioxidant defense mechanisms. Repeated exposure to moderate oxidative stimuli can stimulate upregulation of antioxidant enzymes and activation of cellular repair systems, ultimately improving the organism’s resistance to oxidative damage (3, 5).

Hyperbaric oxygen (HBO) therapy has been widely used over the past several decades both in experimental research and clinical practice. This therapeutic approach involves intermittent exposure to 100% oxygen at pressures higher than normal atmospheric pressure. Under such conditions, the amount of dissolved oxygen in plasma increases exponentially, enhancing oxygen delivery to tissues. In addition to its clinical applications, HBO exposure has been shown to influence multiple cellular processes, including gene expression, signal transduction pathways, and apoptotic mechanisms (6). Experimental studies suggest that HBO preconditioning can modulate oxidative stress responses by inducing adaptive antioxidant mechanisms (7, 8). Intermittent exposure to hyperoxia results in transient increases in ROS, which act as signaling molecules that further trigger cellular defense pathways. This adaptive response may lead to increased expression and activity of antioxidant enzymes, thereby improving the organism’s capacity to cope with oxidative challenges. The timing and duration of HBO exposure appear to play an important role in this process. In particular, the period between exposures, often described as a “pseudohypoxic” phase, is considered a key stimulus for activating antioxidant defense systems (7, 9, 10).

In addition to oxidative stress parameters, the balance of essential macro- and trace elements in tissues is important for maintaining cellular redox homeostasis. Many trace elements are structural components or cofactors of antioxidant enzymes and are involved in numerous metabolic and signaling pathways (11, 12). Elements such as iron, copper, zinc, and selenium participate in redox reactions and contribute to the activity of enzymes involved in ROS detoxification (13−15). Alterations in tissue concentrations of these elements may influence the oxidative status of cells and tissues (11, 12). Therefore, examining both oxidative stress markers and tissue element levels may help to better understand the physiological responses to metabolic stress.

Considering the complex interactions between oxidative stress, PA, HBO exposure, and trace element homeostasis, further investigation of these mechanisms remains of considerable scientific interest. Understanding how acute physical exercise affects oxidative balance in previously untrained organisms, as well as whether HBO preconditioning can modulate these responses, may contribute to improved knowledge of physiological adaptation to stress and the optimization of training or therapeutic strategies. The aim of this study was to investigate the effects of HBO preconditioning on oxidative stress parameters and antioxidant defense mechanisms following acute PA in previously untrained animals. In addition, the study evaluated the concentrations of macro and trace elements in liver and kidney tissues in order to better characterize the relationship between oxidative status and elemental homeostasis under these experimental conditions.

## 2 Materials and methods

### 2.1 Ethics statement

All experimental procedures were approved by the Ethical Committee for the Protection of Animal Welfare of the Faculty of Medicine, University of Belgrade, and by the Veterinary Directorate of the Ministry of Agriculture, Forestry and Water Management of the Republic of Serbia (approval number: 001422251 2024 14,841 002001323022). The study was conducted in accordance with national regulations governing the use of experimental animals and with established guidelines for laboratory animal care.

### 2.2 Experimental animals

For this study male Wistar rats were used. The animals were bred at the Institute for Medical Research, University of Belgrade, Serbia. At the start of the experiment, the animals were 16 weeks old with an average body mass of approximately 300 g. Animals were housed under controlled laboratory conditions at a temperature of 22 ± 1 °C and relative humidity of 65 ± 1%, with a 12 h light/dark cycle. Rats were kept in standard laboratory cages and provided with a commercial laboratory diet (Veterinarski zavod Subotica AD, Serbia) and water *ad libitum* throughout the study.

### 2.3 Experimental design and experimental protocol

Following an acclimatization period under standard housing conditions, the animals were randomly divided into four experimental groups: control (Control, *n* = 8), physical activity (PA, *n* = 8), hyperbaric oxygenation (HBO, *n* = 8), and PA following HBO exposition (HBO + PA, n = 8). Animals in the PA group were subjected to a single bout of swimming exercise lasting for 60 min. Swimming was performed individually in cylindrical containers (depth 50 cm, diameter 30 cm) filled with water maintained at 37 °C in order to minimize thermal stress during the exercise protocol (16). Animals assigned to HBO treatment were exposed to 100% oxygen in a custom made experimental hyperbaric chamber. The HBO protocol consisted of a gradual compression phase lasting 10 min, followed by exposure at 2.0 atmospheres absolute (ATA) for 60 min, and a subsequent decompression period of 10 min. The treatment was performed twice daily at 12-h intervals over a period of two consecutive days. The final HBO exposure was conducted 12 h prior to animal sacrifice (17, 18). Animals in the combined treatment group (HBO + PA) underwent the same HBO exposure protocol and were subsequently subjected to the swimming exercise session previously described. In contrast, animals in the HBO group were exposed only to hyperbaric oxygen without subsequent physical activity. The described HBO procedure did not induce visible stress responses in the animals and therefore did not require sedation. At the end of the experimental protocol, animals were anesthetized with sodium pentobarbital (35 mg/kg body weight, intraperitoneally). Blood samples were collected by cardiac puncture into tubes containing lithium heparin for further analysis. After blood collection, animals were euthanized by administration of a lethal dose of sodium pentobarbital (150 mg/kg body weight, intraperitoneally). Immediately following euthanasia, liver and kidney tissues were excised, rinsed in cold physiological saline, and processed for subsequent biochemical and elemental analyses. Animals subjected to the physical activity protocol were sacrificed immediately after completion of the exercise session, whereas animals that were not exposed to physical activity were sacrificed at the corresponding time point to ensure comparable experimental conditions among groups.

### 2.4 Hematological and biochemical analyses

Hematological parameters were determined after blood collection using an automated hematology analyzer (CELLTAC G MEK-9100, Nihon Kohden, Tokyo, Japan). The following parameters were assessed: red blood cell count (RBC), hemoglobin concentration (HGB), hematocrit (HCT), mean corpuscular volume (MCV), mean corpuscular hemoglobin (MCH), mean corpuscular hemoglobin concentration (MCHC), total leukocyte count (WBC), lymphocyte count (LI), granulocyte count (GRA), monocyte count (MO), and platelet count (PLT).

For subsequent biochemical and enzymatic analyses, blood samples were centrifuged at 4000 rpm for 20 min (Heraeus Megafuge 1.0 R, Heraeus, Hanau, Germany) to separate plasma from erythrocytes. The erythrocyte pellet was washed three times with physiological saline and centrifuged for 10 min at 2000 rpm during each washing step to remove residual plasma components. The washed erythrocytes were used for determination of antioxidant enzyme activities. Plasma obtained after centrifugation was used for subsequent biochemical analyses. Biochemical parameters for liver and kidney function were measured using an automated biochemical analyzer (COBAS INTEGRA 400 plus, Hoffmann-La Roche, Basel, Switzerland). The following parameters were determined: alanine aminotransferase (ALT), aspartate aminotransferase (AST), total protein (PRO), albumin (ALB), creatinine (CRE), and blood urea nitrogen (BUN).

### 2.5 Determination of oxidative stress and antioxidant defense parameters in plasma

#### 2.5.1 Lipid peroxidation in plasma

The degree of lipid peroxidation in plasma was assessed by determining the concentration of thiobarbituric acid reactive substances (TBARS). The assay is based on the reaction of malondialdehyde (MDA), a secondary product of lipid peroxidation, with thiobarbituric acid (TBA), resulting in the formation of a colored complex measurable spectrophotometrically. Plasma samples were mixed with trichloroacetic acid (TCA) to precipitate proteins and incubated on ice for 10 min. Following centrifugation, the obtained supernatant was reacted with thiobarbituric acid and incubated in a boiling water bath for 15 min to allow formation of the MDA-TBA complex. After cooling, absorbance was measured spectrophotometrically at 540 nm. The concentration of TBARS was expressed as nmol per mL of plasma (19).

#### 2.5.2 Determination of antioxidant enzyme activities in erythrocytes

The activities of antioxidant enzymes in erythrocytes were determined spectrophotometrically using erythrocyte lysates. Prior to enzyme activity measurements, erythrocytes were lysed with deionized water. The hemoglobin content in erythrocyte lysates was determined using Drabkin’s reagent in order to normalize enzyme activities. Briefly, erythrocyte lysate was mixed with Drabkin’s reagent and incubated at room temperature in the dark for 15 min. Absorbance was then measured at 545 nm, and hemoglobin concentration was calculated according to the established conversion factor (20).

#### 2.5.3 Superoxide dismutase activity

Superoxide dismutase (SOD) activity was determined using a spectrophotometric method based on the inhibition of adrenaline auto-oxidation. In alkaline conditions, adrenaline undergoes spontaneous oxidation, producing adrenochrome, which can be monitored spectrophotometrically. The presence of SOD inhibits this reaction by scavenging superoxide radicals. The reaction mixture consisted of carbonate buffer containing EDTA and adrenaline solution prepared in hydrochloric acid. The rate of adrenochrome formation was monitored by measuring the change in absorbance at 480 nm. Enzyme activity was expressed as units per gram of hemoglobin (U/g Hb) (21).

#### 2.5.4 Catalase activity

Catalase (CAT) activity in erythrocyte lysates was determined by monitoring the rate of hydrogen peroxide decomposition. The method is based on measuring the decrease in absorbance of hydrogen peroxide at 230 nm. The reaction mixture contained TRIS/EDTA buffer and hydrogen peroxide solution, to which an appropriate volume of erythrocyte lysate was added. The change in absorbance was recorded spectrophotometrically over a three-minute interval at 30-s time points. Catalase activity was expressed as kilo units per gram of hemoglobin (KU/g Hb) (22).

#### 2.5.5 Glutathione reductase activity

Glutathione reductase (GR) activity was performed using a method based on monitoring the oxidation of NADPH at 340 nm, which occurs during the reduction of GSSG (23). The change in absorbance was monitored over 3 min at 340 nm, and enzyme activity was expressed in U/g Hb (23).

### 2.6 Determination of oxidative stress markers in liver and kidney tissue

Liver and kidney tissues were homogenized in ice-cold Tris–EDTA buffer (0.05 M, pH 7.4) using a tissue homogenizer. The homogenates were subsequently centrifuged, and supernatants were used for determination of oxidative stress parameters. The degree of lipid peroxidation in tissue samples was assessed by measuring malondialdehyde (MDA) concentration using the thiobarbituric acid reactive substances (TBARS) assay (24).

Total sulfhydryl (–SH) group content was determined using Ellman’s reagent (5,5′-dithiobis-2-nitrobenzoic acid, DTNB). Non-protein sulfhydryl groups were determined after protein precipitation with sulfosalicylic acid, while protein sulfhydryl group levels were calculated as the difference between total and non-protein sulfhydryl groups (25).

### 2.7 Determination of macro-, micro-, and trace elements in tissue samples

Elemental analysis of liver and kidney tissue samples was performed using inductively coupled plasma mass spectrometry (ICP-MS). Tissue samples were thawed at 4 °C and weighed. Approximately 0.5 g of each sample was subjected to microwave-assisted acid digestion using concentrated nitric acid and hydrogen peroxide in Teflon digestion vessels. The digestion procedure was carried out in a microwave digestion system according to a temperature-controlled program. After digestion, the obtained solutions were quantitatively transferred to volumetric flasks and diluted with deionized water to a final volume suitable for instrumental analysis. Element concentrations were determined using an ICP-MS instrument (iCAP Q, Thermo Scientific, Bremen, Germany). Calibration curves were prepared using multielement standard solutions, while internal standards were applied during measurements to ensure analytical accuracy and instrument stability. The concentrations of macroelements (Na, K, Ca, Mg, and P), essential trace elements (Fe, Zn, Cu, Mn, Se, and Co), and non-essential trace elements (As, Cd, Hg, and Pb) were quantified in tissue samples. The obtained results were expressed relative to the tissue mass (26).

### 2.8 Statistical analysis

Statistical analyses were performed using GraphPad Prism for Windows (version 9.0, GraphPad Software Inc., La Jolla, CA, USA). Results are presented as mean ± standard deviation (SD). The value of *p* < 0.05 was considered statistically significant. The normality of data distribution was assessed using the Shapiro–Wilk test. For datasets showing normal distribution, differences among experimental groups were evaluated using two-way analysis of variance (ANOVA) followed by Tukey’s *post hoc* multiple comparison test. In addition to *p* values, effect sizes were estimated using partial eta squared (ηp^2^) to assess the magnitude of the observed effects.

## 3 Results

### 3.1 Hematological parameters

Complete blood count parameters are presented in Table 1. Two-way ANOVA showed no significant interaction between HBO and PA on RBC count (*p* = 0.1643; ηp^2^ = 0.067). Likewise, PA did not exert a significant effect (*p* = 0.3258; ηp^2^ = 0.033), whereas HBO had a statistically significant effect (*p* = 0.0059; ηp^2^ = 0.235), with higher RBC values observed in HBO-treated groups. *Post hoc* Tukey analysis indicated significant differences between the PA and HBO groups (*p* < 0.05), as well as between the PA and HBO + PA groups (p < 0.05). A similar pattern was observed for hemoglobin levels. No significant interaction between HBO and PA was detected (*p* = 0.2029; ηp^2^ = 0.056), and PA showed no significant main effect (*p* = 0.5731, ηp^2^ = 0.011). In contrast, HBO had a significant effect (*p* = 0.0023; ηp^2^ = 0.277). *Post hoc* analysis revealed significant differences between the PA and HBO groups (*p* < 0.05), and between the PA and HBO + PA groups (p < 0.05). For hematocrit values, two-way ANOVA also revealed no significant interaction between HBO and PA (*p* = 0.1289; ηp^2^ = 0.078). PA did not affect hematocrit (*p* = 0.4395, ηp^2^ = 0.021), whereas HBO had a significant effect (*p* = 0.0077; ηp^2^ = 0.221). *Post hoc* Tukey analysis showed a significant difference only between the PA and HBO + PA groups (*p* < 0.05). No significant interactions or main effects were observed for MCV, MCH, or MCHC values.

**Table 1:** Complete blood count parameters.

Parameter	Control (*n* = 8)	PA (*n* = 8)	HBO (*n* = 8)	HBO + PA (*n* = 8)
RBC (10^12^/L)	9.54 ± 0.47	8.84 ± 1.57	10.00 ± 0.33^**#**^	10.13 ± 0.33^**#**^
HBG (g/L)	146.80 ± 9.51	138.30 ± 23.11	155.60 ± 4.53^**#**^	158.90 ± 5.03^**#**^
HCT (%)	43.44 ± 2.12	40.64 ± 6.76	45.26 ± 1.50	46.25 ± 1.38^**#**^
MCV (fL)	45.53 ± 0.75	45.81 ± 1.30	45.28 ± 0.52	45.64 ± 0.50
MCH (pg)	15.36 ± 0.30	15.68 ± 0.52	15.55 ± 0.34	15.68 ± 0.21
MCHC (g/L)	337.06 ± 8.02	341.30 ± 9.78	344.00 ± 4.72	343.90 ± 1.73
WBC (10^9^/L)	5.13 ± 1.78	4.22 ± 1.72	3.95 ± 0.89	3.14 ± 0.73^*^
LI (10^9^/L)	3.22 ± 0.94	2.25 ± 1.13	2.02 ± 0.52^*^	1.54 ± 0.47^**^
GRA (10^9^/L)	1.21 ± 0.47	1.02 ± 0.41	1.20 ± 0.55	0.89 ± 0.23
MO (10^9^/L)	0.97 ± 0.49	0.95 ± 0.37	0.72 ± 0.33	0.71 ± 0.19
PLT (10^3^/μL)	315.20 ± 189.50	372.50 ± 165.90	404.10 ± 107.10	417.50 ± 62.12

**p < 0.01, *p < 0.05 compared to control group, #*p* < 0.05 compared to PA group. RBC, red blood cells; HGB, hemoglobin; HCT, hematocrit; MCV, mean corpuscular volume; MCH, mean corpuscular hemoglobin; MCHC, mean corpuscular hemoglobin concentration; WBC, white blood cells; LI, lymphocytes; GRA, granulocytes; MO, monocytes; PLT, platelets.

Regarding WBC levels, no significant interaction between HBO and PA was found (*p* = 0.9215; ηp^2^ = 0.0003). PA did not show a significant main effect (*p* = 0.0847; ηp^2^ = 0.099), whereas HBO significantly influenced WBC count (*p* = 0.0255; ηp^2^ = 0.161), with lower values in HBO-treated groups. *Post hoc* analysis demonstrated a significantly reduced WBC count in the HBO + PA group compared to the control group (p < 0.05). In the case of lymphocyte count, no significant interaction between HBO and PA was observed (*p* = 0.6399; ηp^2^ = 0.008). However, both PA (*p* = 0.0453; ηp^2^ = 0.131) and HBO (*p* = 0.0082; ηp^2^ = 0.218) exhibited significant main effects. *Post hoc* Tukey analysis identified a significant difference between the control and HBO (*p* < 0.05), and HBO + PA groups (*p* < 0.01). Finally, no significant interactions or main effects were detected for GRA, MO, or PLT values.

### 3.2 Biochemical parameters of liver and kidney function

To assess liver and kidney function, selected biochemical parameters were evaluated (Table 2). Regarding AST levels, two-way ANOVA revealed no significant effects of HBO, PA, or their interaction, (interaction: *p* = 0.4186; ηp^2^ = 0.029; PA: *p* = 0.6313; ηp^2^ = 0.010; HBO: *p* = 0.1770; ηp^2^ = 0.078). In contrast, a significant main effect of HBO on ALT activity was observed (*p* = 0.0071; ηp^2^ = 0.275), whereas neither PA (*p* = 0.8731; ηp^2^ = 0.001) nor interaction (*p* = 0.1545; ηp^2^ = 0.086) reached statistical significance. *Post hoc* analysis identified a significant difference between the PA and HBO groups (*p* < 0.05). For total protein levels, no significant interaction between HBO and PA was detected (*p* = 0.7380; ηp^2^ = 0.005), and PA showed no significant main effect (*p* = 0.7610; ηp^2^ = 0.004). However, HBO exerted a statistically significant effect (*p* < 0.0001; ηp^2^ = 0.574). A similar pattern was observed for albumin levels, with no significant interaction (*p* = 0.4109; ηp^2^ = 0.030) and no effect of PA (*p* = 0.6282; ηp^2^ = 0.010), while HBO again demonstrated a highly significant main effect (*p* < 0.0001; ηp^2^ = 0.704). *Post hoc* analysis confirmed that both total protein and albumin levels were significantly elevated in HBO-treated groups compared to both control and PA groups (total protein: HBO vs. control and vs. PA, *p* < 0.01; HBO + PA vs. control and vs. PA, p < 0.01; albumin: HBO vs. control and vs. PA, *p* < 0.001; HBO + PA vs. control and vs. PA, p < 0.001). No significant effects of HBO, PA, or their interaction were observed for creatinine levels (interaction: *p* = 0.5242; ηp^2^ = 0.018; PA: *p* = 0.0584; ηp^2^ = 0.147; HBO: *p* = 0.7016; ηp^2^ = 0.007). In contrast, BUN levels were significantly affected by HBO (*p* = 0.0003; ηp^2^ = 0.436), while neither PA (*p* = 0.6665; ηp^2^ = 0.008) nor the interaction (*p* = 0.6322; ηp^2^ = 0.010) showed significant effects. BUN levels were elevated in the HBO group (vs. PA group, *p* < 0.05) and in the HBO + PA group (vs. control, *p* < 0.05; vs. PA group, p < 0.05).

**Table 2:** Biochemical parameters for liver and kidney function assessment.

Parameter	Control (*n* = 6)	PA (*n* = 7)	HBO (*n* = 7)	HBO + PA (*n* = 7)
AST (U/L)	106.30 ± 11.79	112.30 ± 23.98	108.10 ± 10.46	104.10 ± 8.90
ALT (U/L)	72.00 ± 7.65	65.64 ± 6.20	82.87 ± 16.50^**#**^	77.77 ± 5.54
PRO (g/L)	69.83 ± 2.98	69.11 ± 3.16	75.68 ± 2.78^**,##^	75.64 ± 2.59^**,##^
ALB (g/L)	41.03 ± 2.49	40.01 ± 2.62	46.33 ± 1.51^***,###^	46.07 ± 0.96^***,###^
CRE (μmol/L)	28.67 ± 4.46	27.86 ± 3.13	31.14 ± 3.08	29.29 ± 2.50
BUN (mmol/L)	6.60 ± 0.45	6.59 ± 0.64	7.07 ± 1.11^**#**^	7.97 ± 0.66^*,#^

***p < 0.001, **p < 0.01, *p < 0.05 compared to control group, ###*p* < 0.001, ##*p* < 0.01, #*p* < 0.05 compared to PA group. AST, aspartate aminotransferase; ALT, alanine aminotransferase; PRO, total protein; ALB, albumin; CRE, creatinine; BUN, blood urea nitrogen. Sample sizes varied (*n* = 6–7) as a result of insufficient sample volume for specific measurements.

### 3.3 Oxidative stress parameters in blood, kidney and liver tissue

**Figure 1:**
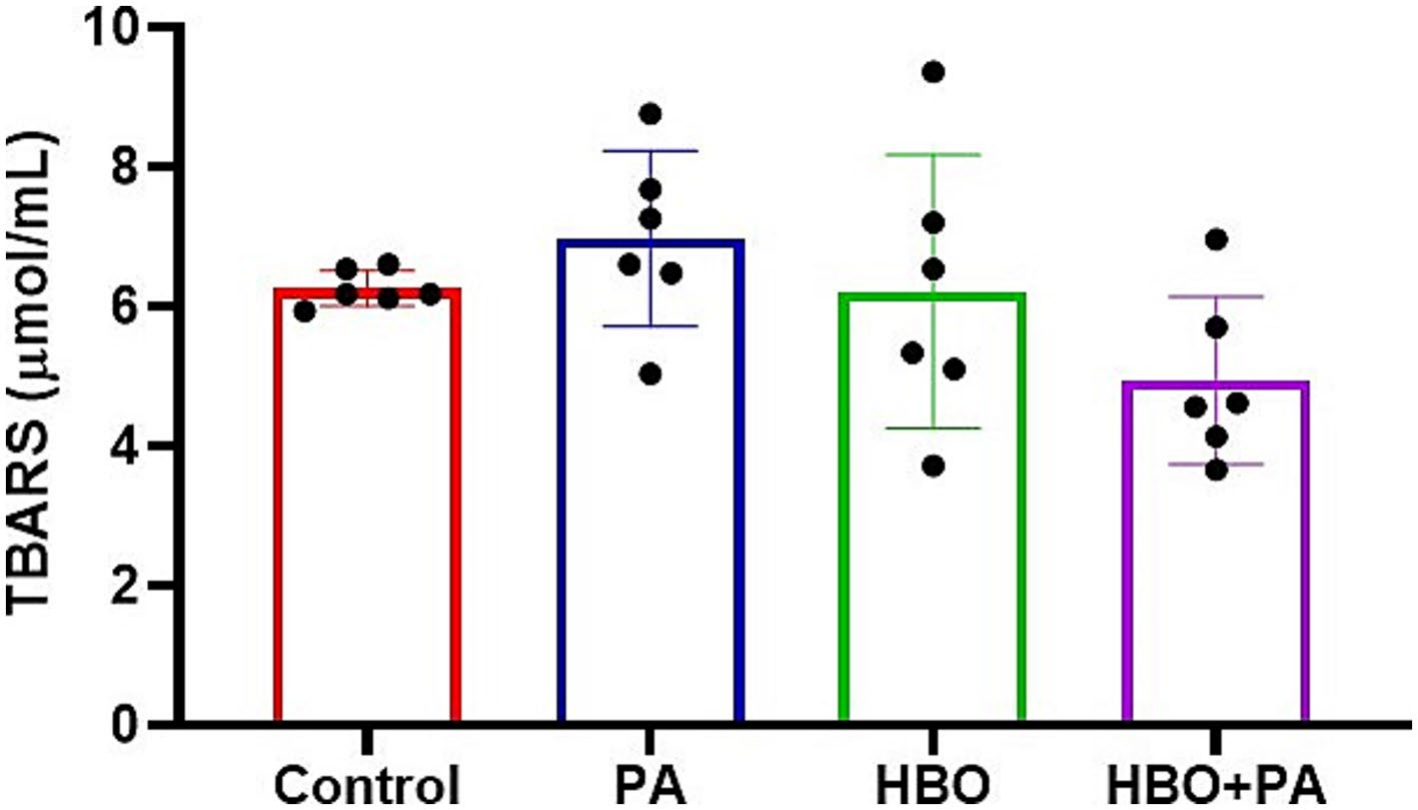
Plasma lipid peroxidation levels in experimental groups. TBARS, thiobarbituric acid reactive substances. Sample sizes varied (*n* = 6) as a result of insufficient sample volume for specific measurements.

**Figure 2:**
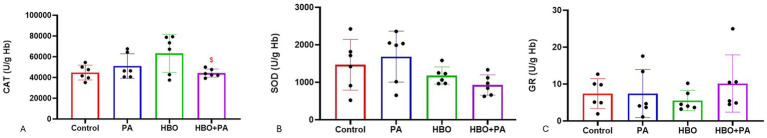
Catalase (CAT; **A**), superoxide dismutase (SOD; **B**), glutathione reductase (GR; **C**) activities in erythrocytes across the experimental groups. $*p* < 0.05 compared to HBO group. Sample sizes varied (*n* = 6) as a result of insufficient sample volume for specific measurements.

Two-way ANOVA revealed no significant effects of HBO, PA, or their interaction (*p* = 0.6078; ηp^2^ = 0.013) on plasma TBARS levels, however, both PA (*p* = 0.0801; ηp^2^ = 0.145) and HBO (*p* = 0.0671; ηp^2^ = 0.158) approached statistical significance (PA vs. PA + HBO, p = 0.06; Figure 1). For CAT activity, a significant interaction between PA and HBO was detected (*p* = 0.0160; ηp^2^ = 0.257), whereas neither PA (*p* = 0.1976; ηp^2^ = 0.081) nor HBO (*p* = 0.2392; ηp^2^ = 0.069) showed significant main effects. Specifically, CAT activity increased in HBO-treated sedentary animals but decreased in HBO-treated physically active animals. *Post hoc* analysis revealed a significant difference between the HBO and PA + HBO groups (*p* < 0.05) (Figure 2A). In contrast, SOD activity was significantly affected by HBO (*p* = 0.0212; ηp^2^ = 0.238), whereas PA had no significant effect (*p* = 0.9336; ηp^2^ = 0.0004), and no significant interaction was observed (*p* = 0.2785; ηp^2^ = 0.058). Additionally, no significant differences were detected between individual experimental groups (Figure 2B).

Finally, GR activity was not significantly influenced by HBO, PA, or their interaction (interaction: *p* = 0.3274; ηp^2^ = 0.048; PA: *p* = 0.3267; ηp^2^ = 0.048; HBO: *p* = 0.8582; ηp^2^ = 0.002; Figure 2C).

**Figure 3:**
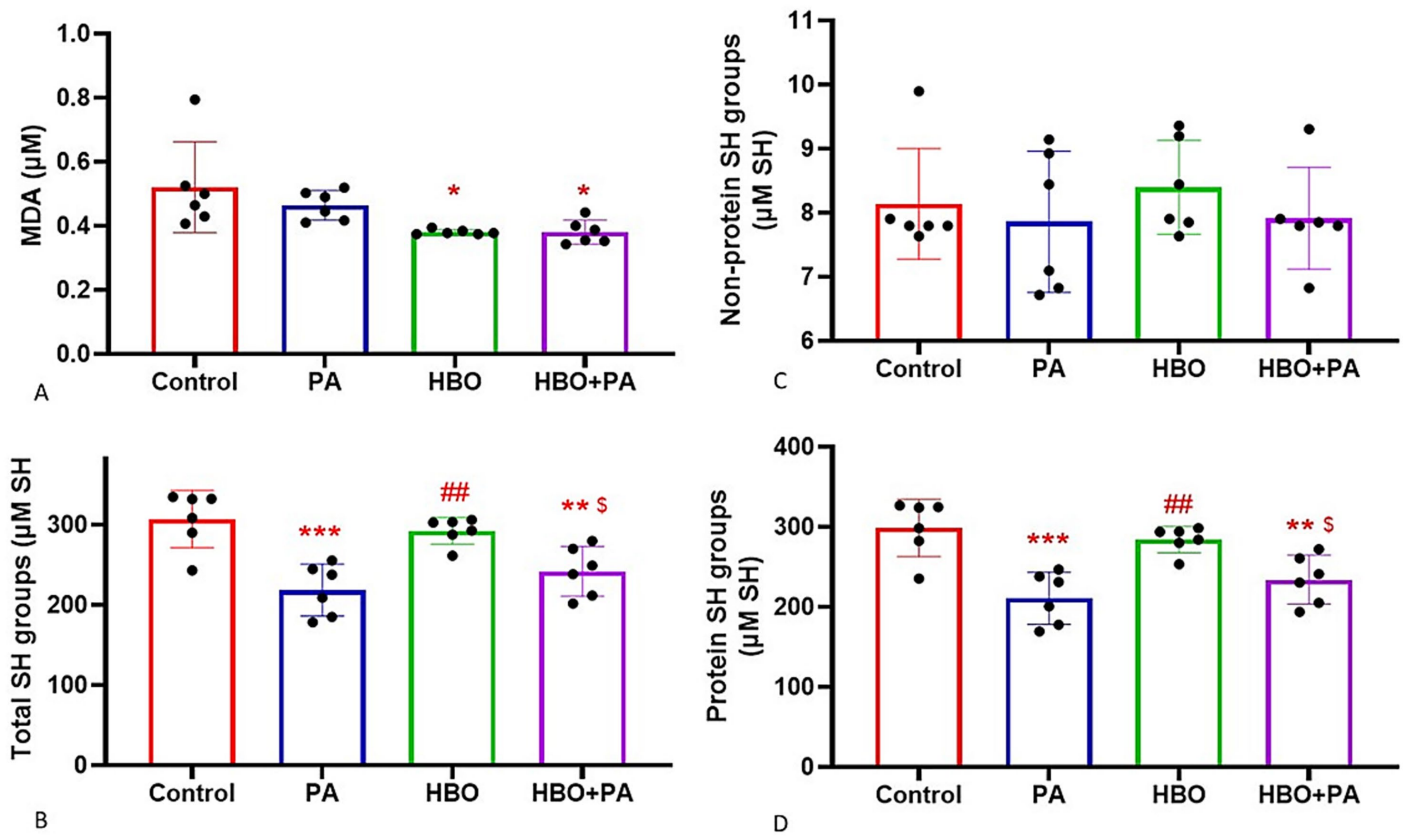
Malondialdehyde (MDA; **A**), total SH groups **(B)**, non-protein SH groups, **(C)** protein SH groups, **(D)** concentration in kidney tissue in experimental groups. ^*^p < 0.05, ^**^p < 0.01, ***p < 0.001, compared to control group, ##*p* < 0.01 compared to PA group, $*p* < 0.05 compared to HBO group. Sample sizes varied (*n* = 6) as a result of insufficient sample volume for specific measurements.

In kidney tissue, a significant main effect of HBO on MDA levels was observed (*p* = 0.0020; ηp^2^ = 0.388), whereas no significant effects of PA (*p* = 0.3775; ηp^2^ = 0.039) or their interaction (*p* = 0.3864; ηp^2^ = 0.038) were detected. *Post hoc* analysis revealed a significant reduction in lipid peroxidation in both HBO-treated groups compared to the control group (HBO vs. control, *p* < 0.05; HBO + PA vs. control, p < 0.05; Figure 3A). Two-way ANOVA demonstrated a significant main effect of PA on total SH levels in kidney tissue (*p* < 0.0001; ηp^2^ = 0.619), while no significant effects of HBO (*p* = 0.7169; ηp^2^ = 0.007) or interaction between the factors (*p* = 0.1380; ηp^2^ = 0.107) were observed. Total SH levels were significantly reduced in the PA group compared to controls (*p* < 0.001), with a similar decrease observed in the HBO + PA group (*p* < 0.01). In contrast, total SH levels in the HBO group were significantly higher than in both the PA group (p < 0.01) and the HBO + PA group (*p* < 0.05), remaining comparable to the control group (Figure 3B). Further analysis revealed no significant effects of PA, HBO, or their interaction on non-protein SH levels in kidney tissue (Figure 3C). In contrast, protein SH levels in kidney tissue were significantly affected by PA (p < 0.0001; ηp^2^ = 0.618), with no significant effect of HBO (*p* = 0.7255; ηp^2^ = 0.006) or interaction (*p* = 0.1348; ηp^2^ = 0.108). *Post hoc* comparisons showed patterns consistent with those observed for total SH levels (PA vs. control, *p* < 0.001; PA vs. HBO, p < 0.01; HBO + PA vs. control, p < 0.01; HBO + PA vs. HBO, *p* < 0.05; Figure 3D).

**Figure 4:**
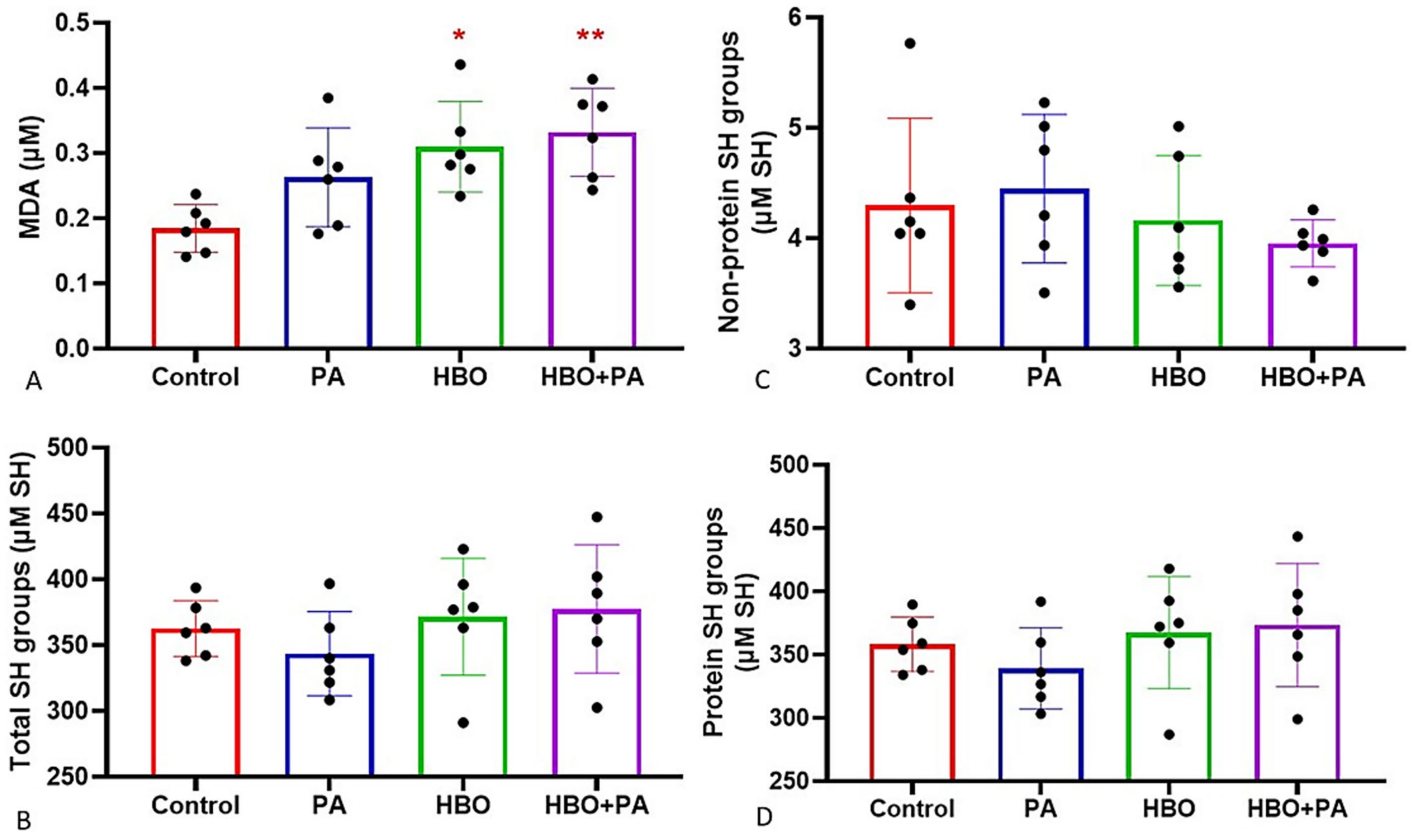
Malondialdehyde (MDA; **A**), total SH groups **(B)**, non-protein SH groups, **(C)** protein SH groups, **(D)** concentration in liver tissue in experimental groups. *p < 0.05, **p < 0.01. Sample sizes varied (*n* = 6) as a result of insufficient sample volume for specific measurements.

In liver tissue, a significant main effect of HBO (*p* = 0.0014; ηp^2^ = 0.408) on MDA levels was observed, whereas neither PA (*p* = 0.0697; ηp^2^ = 0.155) nor the interaction between the factors (*p* = 0.2928; ηp^2^ = 0.055) reached statistical significance. *Post hoc* analysis indicated that both the HBO and HBO + PA groups exhibited significantly higher values compared to the control group (*p* < 0.05 and *p* < 0.01, respectively, Figure 4A). No significant effects of PA, HBO, or their interaction were found for total SH, non-protein SH, or protein SH levels in liver tissue (Figures 4B–D).

### 3.4 Element concentrations in kidney and liver tissue

Considering the concentrations of macroelements in kidney tissue (Table 3), significant main effects of both HBO (*p* = 0.0005; ηp^2^ = 0.345) and PA (*p* = 0.0137; ηp^2^ = 0.192) on sodium levels were observed, without a significant interaction between these factors (*p* = 0.6798; ηp^2^ = 0.006). Sodium levels were reduced in both HBO-treated and physically active groups. *Post hoc* analysis showed that the control group had significantly higher sodium levels compared to the HBO (*p* < 0.05) and PA + HBO groups (*p* < 0.001). Furthermore, HBO exerted significant effects on potassium (*p* = 0.0024; ηp^2^ = 0.278), magnesium (*p* = 0.0025; ηp^2^ = 0.274), and phosphorus (*p* = 0.0073; ηp^2^ = 0.223) levels. HBO treatment, alone or combined with physical activity, resulted in significantly lower potassium levels (HBO vs. control and PA, *p* < 0.01), as well as reduced magnesium and phosphorus levels (for both elements: HBO vs. control and PA, p < 0.05).

**Table 3:** Macro- and trace element concentrations in kidney tissue.

Parameter	Control (*n* = 8)	PA (*n* = 8)	HBO (*n* = 8)	HBO + PA (*n* = 8)
Na (mg/kg)	1525.00 ± 166.80	1354.00 ± 141.30	1282.00 ± 135.50^*^	1158.00 ± 193.80^***^
K (mg/kg)	3405.00 ± 352.00	3293.00 ± 285.00	2755.00 ± 244.10^**, ##^	3042.00 ± 586.20
Ca (mg/kg)	83.82 ± 18.72	88.99 ± 12.09	67.62 ± 14.64	78.42 ± 28.06
Mg (mg/kg)	219.30 ± 23.27	221.60 ± 18.91	179.20 ± 19.46^*, #^	202.50 ± 37.00
P (mg/kg)	2997.00 ± 306.80	3010.00 ± 268.50	2532.00 ± 279.90^*, #^	2782.00 ± 482.80
Fe (mg/kg)	75.80 ± 8,68	53.53 ± 12.10^***^	68.01 ± 9.41 ^**#**^	60.92 ± 8.06^*^
Zn (mg/kg)	27.49 ± 3.02	25.30 ± 1.39	22.15 ± 1.52^*^	23.91 ± 4.55
Cu (mg/kg)	16.61 ± 2.95	18.45 ± 3.62	19.01 ± 3.58	20.16 ± 4.42
Mn (mg/kg)	0.94 ± 0.15	0.94 ± 0.08	0.83 ± 0.16	0.88 ± 0.20
Se (mg/kg)	1.14 ± 0.19	0.98 ± 0.15	1.11 ± 0.18	1.10 ± 0.29
Co (mg/kg)	0.13 ± 0.02	0.14 ± 0.02	0.14 ± 0.02	0.14 ± 0.03
As (μg/kg)	144.70 ± 34.09	76.50 ± 26.39^***^	135.80 ± 25.79 ^**##**^	73.96 ± 37.07^***, $$^
Cd (μg/kg)	14.19 ± 13.93	13.36 ± 8.63	12.85 ± 14.86	9.86 ± 4.76
Hg (μg/kg)	9.54 ± 2.85	4.76 ± 3.27^**^	5.03 ± 1.88^**^	4.94 ± 1.54^**^
Pb (μg/kg)	18.14 ± 12.78	20.36 ± 10.10	10.86 ± 3.80	9.96 ± 4.99

*p < 0.05, **p < 0.01, ***p < 0.001, compared to control group, #*p* < 0.05, ##*p* < 0.01, compared to PA group, $$*p* < 0.01 compared to HBO group. For Cd *n* = 8 in control and *n* = 7 in PA group. For Hg *n* = 7 in HBO+PA group. Na, sodium; K, potassium; Ca, calcium; Mg, magnesium; P, phosphorus; Fe, iron; Zn, zinc; Cu, copper; Mn, manganese; Se, selenium; Co, cobalt; As, arsenic; Cd, cadmium; Hg, mercury; Pb, lead.

Regarding trace elements, iron levels were influenced by a significant interaction between HBO and PA (*p* = 0.0318; ηp^2^ = 0.149), as well as a significant main effect of PA (*p* = 0.0001; ηp^2^ = 0.396). The lowest iron concentrations were observed in physically active groups (PA vs. control, *p* < 0.001; PA vs. HBO, p < 0.05; PA + HBO vs. control, p < 0.05). For zinc, a significant effect of HBO was found (*p* = 0.0026; ηp^2^ = 0.273), while neither PA (*p* = 0.8326, ηp^2^ = 0.002) nor the interaction (*p* = 0.0625; ηp^2^ = 0.115) reached significance. Zinc levels were lowest in the HBO group, with a significant reduction compared to control (*p* < 0.05). Among non-essential trace elements, PA showed a significant main effect on arsenic levels (*p* < 0.0001; ηp^2^ = 0.550), whereas HBO significantly affected lead levels (*p* = 0.0080; ηp^2^ = 0.219), with lower concentrations observed in HBO-treated groups. Arsenic levels were lowest in physically active groups, specifically in the PA group compared to control (*p* < 0.001) and HBO (*p* < 0.01), as well as in the PA + HBO group compared to control (*p* < 0.001) and HBO (*p* < 0.01). Additionally, mercury levels were influenced by HBO (*p* = 0.0229; ηp^2^ = 0.172), PA (*p* = 0.0112; ηp^2^ = 0.208), and their interaction (*p* = 0.0144; ηp^2^ = 0.196), with all treated groups showing significantly lower levels compared to control (control vs. PA, *p* < 0.01; vs. HBO, *p* < 0.01; vs. PA + HBO, p < 0.01).

In liver tissue (Table 4), significant main effects of both, PA (*p* < 0.0001; ηp^2^ = 0.431) and HBO (*p* = 0.0200; ηp^2^ = 0.173) were observed on sodium levels, without a significant interaction between these factors (*p* = 0.2490; ηp^2^ = 0.046). *Post hoc* analysis indicated that PA led to significantly lower sodium levels compared to control (PA vs. control, *p* < 0.01; HBO + PA vs. control, *p* < 0.001). A significant main effect of HBO was also observed for potassium levels (*p* = 0.0023; ηp^2^ = 0.277), whereas neither PA (*p* = 0.1559; ηp^2^ = 0.068) nor the interaction (*p* = 0.2169; ηp^2^ = 0.064) reached significance. Potassium concentrations were reduced in HBO-treated groups (HBO vs. control, *p* < 0.01; HBO vs. PA, *p* < 0.05). Magnesium levels were significantly affected by PA (*p* = 0.0003; ηp^2^ = 0.368), while no significant contribution of HBO (*p* = 0.2669; ηp^2^ = 0.042) or interaction between factors (*p* = 0.3729; ηp^2^ = 0.027) was detected. *Post hoc* analysis showed that magnesium concentrations were significantly higher in both PA and HBO + PA groups compared to HBO group (*p* < 0.01). Phosphorus levels were influenced by PA (*p* = 0.0252; ηp^2^ = 0.161), while no significant effects of HBO (*p* = 0.3661; ηp^2^ = 0.028) or interaction (*p* = 0.1406; ηp^2^ = 0.073) were observed; however, *post hoc* analysis did not reveal significant pairwise differences between groups. Regarding trace elements, no significant main effects, interactions, or group differences were observed for most elements. Exceptions included manganese (significant main effect of PA, *p* = 0.0431; ηp^2^ = 0.134), copper (significant main effect of PA, *p* = 0.0454; ηp^2^ = 0.131), cobalt (significant main effect of HBO, *p* = 0.0315; ηp^2^ = 0.150), and selenium (significant main effect of HBO, *p* = 0.0250; ηp^2^ = 0.162).

**Table 4:** Macro- and trace element concentrations in liver tissue.

Parameter	Control (*n* = 8)	PA (*n* = 8)	HBO (*n* = 8)	HBO + PA (*n* = 8)
Na (mg/kg)	615.70 ± 46.88	545.70 ± 26.44^**^	572.30 ± 30.20	530.30 ± 27.30^***^
K (mg/kg)	4099.00 ± 208.30	4102.00 ± 194.60	3816.00 ± 142.70^**, #^	3987.00 ± 114.20
Ca (mg/kg)	47.39 ± 9.38	47.36 ± 5.55	42.61 ± 7.35	48.37 ± 7.62
Mg (mg/kg)	230.00 ± 11.65	243.70 ± 11.63	221.20 ± 8.87^**##**^	242.80 ± 16.14^**$$**^
P (mg/kg)	3238.00 ± 172.60	3290.00 ± 168.00	3089.00 ± 96.04	3326.00 ± 238.70
Fe (mg/kg)	137.10 ± 21.10	120.40 ± 26.03	134.00 ± 8.93	136.60 ± 9.48
Zn (mg/kg)	26.68 ± 2.34	28.96 ± 2.13	27.74 ± 1.18	29.85 ± 3.27
Cu (mg/kg)	3.49 ± 0.29	3.68 ± 0.24	3.37 ± 0.18	3.57 ± 0.32
Mn (mg/kg)	2.67 ± 0.20	2.78 ± 0.22	2.52 ± 0.21	2.73 ± 0.26
Se (mg/kg)	0.56 ± 0.16	0.61 ± 0.15	0.81 ± 0.19	0.70 ± 0.30
Co (mg/kg)	0.03 ± 0.01	0.03 ± 0.01	0.02 ± 0.01	0.02 ± 0.01
As (μg/kg)	88.43 ± 30.93	90.29 ± 26.42	76.88 ± 20.08	85.14 ± 27.27
Pb (μg/kg)	19.83 ± 7.12	9.65 ± 4.89	20.71 ± 16.52	17.59 ± 7.30

**p < 0.01, ***p < 0.001 compared to control group, #*p* < 0.05, ##*p* < 0.01 compared to PA group, $$*p*<0.01 compared to HBO group. The concentrations of cadmium and mercury were below the level of detectability in the experimental groups. Na, sodium; K, potassium; Ca, calcium; Mg, magnesium; P, phosphorus; Fe, iron; Zn, zinc; Cu, copper; Mn, manganese; Se, selenium; Co, cobalt; As, arsenic; Pb, lead.

## 4 Discussion

The results suggest that HBO exerts a more pronounced effect on hematological parameters than PA, with no evidence of interaction between the two across the majority of measured variables. These findings indicate that HBO influenced circulating red blood cell count quantitatively without altering erythrocyte morphology or hemoglobin content per cell. This represents a particularly interesting finding, considering that previous clinical studies have not demonstrated significant effects on these hematological parameters following acute HBO exposure (27, 28). The present findings may be explained by multiple interacting physiological mechanisms. First, observed changes may be attributed to an acute redistribution of erythrocytes resulting from splenic contraction. In mammals, the spleen functions as a physiologically active erythrocyte reservoir that rapidly mobilizes stored red blood cells in response to sympathetic stimulation (29). Hyperbaric hyperoxia has been shown to modulate autonomic balance and vascular tone (30), potentially triggering splenic vasoconstriction and transient erythrocyte release. Such redistribution would increase circulating erythrocyte count and hematocrit without affecting erythrocyte indices, consistent with the unchanged MCV, MCH, and MCHC observed in the present study. Second, HBO is known to induce systemic vasoconstriction through hyperoxia-mediated reductions in nitric oxide bioavailability and increased oxidative signaling (30). Hyperoxia-induced vasoconstriction may reduce plasma volume through vascular fluid shifts and diuresis, leading to relative hemoconcentration. Thus, elevated hematocrit and hemoglobin likely reflect plasma volume contraction rather than true erythrocytosis; however, without plasma volume measurements, differentiation from erythrocyte mobilization is not possible (31, 32). Mihaljević et al. (10) demonstrated that acute hyperbaric oxygen exposure disrupts endothelium-dependent vasorelaxation via oxidative stress and decreased nitric oxide, while intermittent exposure protects vascular function through adaptive antioxidant mechanisms. Notably, in their study, intermittent exposure consisted of 4 daily sessions of 120 min each over 4 consecutive days. Unlike the previous study, our intermittent protocol consisted of four shorter 60-min sessions, spaced over two consecutive days with 12-h intervals, highlighting a different exposure pattern that may influence vascular responses. Despite similar cumulative exposure, this shorter, compressed schedule may elicit distinct hemodynamic and hematological responses. In the present study, the short-term HBO regimen likely activated only transient physiological responses without sufficient time for true erythropoiesis. The concomitant elevations in total protein and albumin may support plasma volume contraction as the underlying mechanism, since acute increases in these proteins are typically attributable to reduced plasma water rather than increased hepatic synthesis. The rise in urea with normal creatinine further suggests a transient pre-renal or dehydration-related effect rather than intrinsic renal impairment. Third, a role for erythropoietin (EPO)-mediated erythropoiesis warrants consideration. Although sustained hyperoxia suppresses hypoxia-inducible factor (HIF)-1α activity and EPO synthesis, intermittent hyperoxic exposure may paradoxically stimulate EPO production during the return to normoxia, a phenomenon described as the “hyperoxic-hypoxic paradox” (33, 34). Repeated HBO sessions could therefore create oscillatory oxygen signaling that functionally mimics hypoxic stimuli at the molecular level, potentially enhancing endogenous EPO production. However, the temporal dynamics of erythropoiesis argue against a substantial contribution of *de novo* red blood cell production in this model. In rats, approximately 3–5 days are typically required for increased EPO levels to translate into detectable reticulocytosis and subsequent elevations in circulating erythrocyte count (35). Given our protocol and the 12-h interval before euthanasia, the exposure may not have been sufficient to induce a detectable erythropoietic response. Importantly, although changes in erythrocyte count, hemoglobin, and hematocrit were observed, oxygen delivery and tissue oxygenation were not directly assessed, no functional evaluation was performed, and erythropoietin levels were not measured; therefore, the proposed mechanisms should be interpreted with caution.

Leukocyte dynamics further support the modulatory role of HBO. The observed decrease in WBC count, particularly in the HBO + PA group, may reflect an anti-inflammatory effect of HBO. Similarly, the reduction in lymphocyte count in both PA and HBO-treated groups suggests that both interventions may influence immune cell distribution or turnover. The selective reduction in lymphocytes, in the absence of changes in other hematological lineages, could suggest that HBO does not exert a generalized suppressive effect on bone marrow function. Rather, this finding may reflect an immunomodulatory effect of HBO, potentially mediated through attenuation of systemic inflammatory signaling, modulation of oxidative stress pathways, or redistribution of lymphocyte subsets. Recent clinical and experimental studies support immunomodulatory effects of HBO (28), including alterations in lymphocyte counts and inflammatory indices such as neutrophil-to-lymphocyte ratio (NLR), and platelet-to-lymphocyte ratio (PLR) (36), which is consistent with our observations of reduced WBC primarily attributable to lymphocyte changes. In addition, transcriptomic analyses in severely ill patients suggest that HBO induces a distinct immune-related gene expression profile, highlighting its potential to influence immune and stress response pathways (37). Furthermore, experimental evidence indicates that HBO modulates key redox-sensitive signaling pathways, including upregulation of HIF-1α and attenuation of NF-κB activity, which may underline both its anti-inflammatory effects and systemic adaptation to repeated oxygen exposure (18). Additionally, acute PA may induce a transient redistribution of immune cells from the circulation to peripheral tissues, reflecting enhanced immune surveillance rather than immune suppression (38). Importantly, the absence of thrombocytopenia and the stability of other leukocyte populations further support the notion that HBO induces specific adaptive hematological responses rather than broad hematopoietic suppression.

The present findings suggest that HBO induces selective, context-dependent modulation of oxidative stress parameters. Although TBARS levels were not significantly altered, the observed tendency toward lower values in HBO-treated groups may indicate a potential reduction in lipid peroxidation. However, overall systemic changes in oxidative stress markers were not clearly pronounced, suggesting that the effects of HBO exposure on redox status remain modest under the applied conditions. Experimental and clinical studies have shown that acute HBO exposure can increase ROS production, whereas intermittent or repeated exposures may induce adaptive antioxidant responses (39–41). In line with this, the present results may reflect an early or transitional phase of redox adaptation rather than a fully established antioxidant response. The significant interaction observed for CAT activity further supports the notion that the antioxidant response to HBO depends on the physiological context, with divergent effects observed in sedentary and physically active animals. Specifically, the increase in CAT activity in sedentary animals following HBO may represent a proactive adaptive response to hyperoxia. In contrast, the reduction of CAT activity in the combined HBO + PA group suggests a different redox dynamics when two oxidative stimuli are present. This interaction may be partially explained by the fact that PA itself can exert a transient pro-oxidant effect (3, 4, 42). Given that animals were sacrificed immediately after PA, it is likely that exercise-induced oxidative processes influenced the observed CAT response, thereby contributing to the interaction between HBO and PA. This suggests that the acute ROS generation triggered by exercise may interfere with or modify the adaptive signaling typically initiated by intermittent HBO. The divergent CAT and SOD responses may further indicate that HBO-induced antioxidant adaptation depends on the underlying physiological and oxidative context, particularly in the presence of exercise-induced redox alterations (43). The fact that HBO demonstrated a significant main effect on SOD activity, independent of exercise, suggests that hyperoxia may act as an important stimulus for shifting the enzymatic antioxidant landscape.

Despite subtle systemic changes, liver tissue showed a significant increase in lipid peroxidation in HBO-exposed groups, indicating localized oxidative stress. Under physiological conditions, the liver is an important site of ROS production due to its metabolic and detoxification activities (44). However, unchanged levels of total protein and non-protein sulfhydryl groups indicate that hepatic antioxidant defense was sufficient to prevent overt oxidative damage, consistent with the mild ALT elevation observed in these animals. On the other hand, in kidney tissue, HBO decreased lipid peroxidation, suggesting a tissue-specific protective or preconditioning effect against oxidative stress. Conversely, PA reduced protein-SH levels, suggesting mild PA-induced oxidative stress and a predominant contribution to renal thiol status in this model, while unchanged non-protein-SH levels may reflect preserved glutathione-mediated antioxidant capacity. The observation of decreased lipid peroxidation in kidney tissue after HBO exposure aligns with recent experimental evidence demonstrating that HBO preconditioning can reduce oxidative damage and enhance antioxidant capacity in renal tissue in animal models of acute injury, suggesting a protective, ROS-modulating effect of HBO in kidney tissue (18). In contrast, the significant increase in liver lipid peroxidation may be attributed to a more pronounced local oxidative response in the liver following HBO exposure, reflecting organ-specific susceptibility to hyperoxic ROS. These tissue-dependent effects are consistent with reports that hyperoxic exposure can produce mild oxidative challenges that trigger adaptive antioxidant responses, a phenomenon related to the hyperoxia-hypoxia paradox described in recent literature (45). Importantly, redox responses may vary across tissues, with localized effects that are not necessarily reflected in circulating biomarkers (46). Since the introduction of HBO therapy into clinical practice in the 1960s, its potential toxic effects have been carefully investigated. These concerns prompted a systematic evaluation of oxygen toxicity in various organs, including the liver. Importantly, most experimental and clinical reports of hyperbaric oxygen toxicity have involved exposure pressures exceeding 3 ATA, where oxygen partial pressures are sufficiently high to overwhelm endogenous antioxidant defenses. In contrast, modern HBOT is typically administered at or below 2 ATA, a range considered therapeutically effective while minimizing the risk of significant oxidative injury. This pressure-dependent effect may explain why, under clinically relevant conditions, oxidative changes are often mild and reversible rather than indicative of overt hepatotoxicity (47).

Our study demonstrates tissue-specific effects of HBO and PA on mineral and trace element concentrations in the kidney and liver. In kidney tissue, HBO exerted consistent main effects on electrolyte concentrations, as evidenced by reduced sodium, potassium, magnesium, and phosphorus levels, independent of PA and without significant interactions. Sodium concentrations were additionally influenced by PA, suggesting partially overlapping but independent physiological responses. Although the underlying mechanisms were not directly assessed, these findings may reflect compartment-specific changes associated with altered renal physiology and redox-sensitive cellular responses during hyperoxic exposure (48–50). Likewise, reductions in renal sodium concentrations following acute PA may be related to exercise-associated shifts in electrolyte balance and renal hemodynamics (51). Trace element concentrations were generally less affected, although iron and zinc demonstrated significant changes. Iron concentrations were predominantly influenced by PA, with lower levels observed in physically active groups and significant interaction between PA and HBO. These findings are in line with previous observations suggesting that intensive physical activity may be associated with reduced iron availability and altered iron distribution (52). Zinc concentrations were lower in HBO-treated animals, which may reflect altered tissue zinc balance under hyperoxic conditions. Since zinc homeostasis is closely linked to oxidative stress and intracellular redox signaling (53, 54), the observed reduction may indicate a localized tissue response to HBO exposure rather than systemic zinc depletion. Non-essential trace elements also exhibited distinct response patterns. Arsenic concentrations were markedly reduced in physically active groups, whereas lead concentrations were lower following HBO treatment. Mercury levels were influenced by both interventions and their interaction, with lower concentrations observed across treated groups. The lower arsenic concentrations observed in PA groups are in agreement with previous reports suggesting that exercise may influence arsenic handling (55, 56). Similarly, lower mercury and lead levels may reflect organ-specific concentration changes associated with physiological responses to HBO and PA, since the kidneys are recognized as a primary target organ for heavy metal toxicity due to their high blood flow and their role in filtering and reabsorbing heavy metals (57). Similarly, a recent study demonstrated a significant effect of HBO therapy in reducing plasma lead concentrations in an experimental model of chronic kidney disease (58). However, these findings should be interpreted cautiously, as only tissue concentrations were assessed. Because plasma, urinary excretion and renal transporter expression were not evaluated, the observed changes should be considered compartment-specific concentration shifts rather than direct evidence of altered transport, detoxification, systemic redistribution, or excretion.

In liver tissue, sodium levels were influenced by both PA and HBO, without a significant interaction, suggesting that each factor contributes separately to the observed reduction. In contrast, potassium levels were selectively affected by HBO, while magnesium levels by PA. It is possible that the observed changes primarily reflect previously described alterations in renal concentrations and the redistribution of these macroelements. Trace element homeostasis in the liver was largely preserved, with only subtle main effects observed for manganese and copper (PA-related) and cobalt and selenium (HBO-related). Such changes may arise from exercise-induced metabolic demand and antioxidant activation, or from hyperoxia-related redox modulation, given their roles in enzymatic and antioxidant systems (59–62).

To the best of our knowledge, a limited number of studies have investigated the effects of acute PA or HBO exposure on macro-, and trace element concentrations in liver and kidney tissues. In this context, the lack of plasma or urinary measurements of these elements represents one of the limitations of the present study. Including such data would have facilitated a more comprehensive interpretation of the results, particularly regarding potential redistribution between compartments. Nevertheless, despite these limitations, the observed findings are intriguing and represent a novel contribution to the field, providing a valuable basis for future research. In addition to the aforementioned, it is important to highlight additional limitations of the study. We acknowledge the issue of multiplicity arising from the large number of endpoints tested. Consequently, certain findings, particularly among the trace element profiles, should be considered exploratory in nature. Additionally, we did not measure erythropoietin, which could have provided insight into the mechanisms underlying the observed increases in red blood cell parameters. Only selected systemic oxidative stress markers were assessed, limiting the understanding of broader oxidative responses and molecular mechanisms. Also, we emphasize a relatively small sample size, limited direct translatability of findings from an acute animal model to human physiology, and differences in the timing of sacrifice between the groups. While animals in the PA group were sacrificed immediately after exercise to capture peak acute metabolic and oxidative changes, the HBO groups were sacrificed 12 h after the final session to allow for the development of adaptive antioxidant responses. This difference, while physiologically justified, introduces a potential confounding factor when comparing acute versus transitional physiological states. The absence of sham pressurization control is another design limitation, as the study cannot fully separate the effects of hyperoxia from those of pressure exposure, chamber confinement, handling, or repeated procedural stress. Despite these constraints, the findings suggest organ-specific redox and trace element responses to short-term HBO exposure and PA.

## 5 Conclusion

Overall, interaction effects between HBO and PA were largely absent across most measured parameters, indicating that their influences were predominantly independent. An exception was observed for catalase activity, which exhibited a significant interaction effect, suggesting selective modulation of antioxidant responses under combined exposure conditions. In contrast, several significant main effects were identified, suggesting HBO as the predominant factor influencing hematological, biochemical, and tissue-specific responses. Specifically, HBO increased erythrocyte count, hemoglobin concentration, and hematocrit without affecting erythrocyte indices and was associated with elevated total protein, albumin, and urea levels. Systemic oxidative stress markers remained largely unchanged; however, pronounced tissue-specific responses were observed. HBO increased lipid peroxidation in liver tissue, whereas in kidney tissue it reduced lipid peroxidation and attenuated PA-associated depletion of sulfhydryl groups, indicating organ-dependent redox modulation. Elemental analysis further indicated that most detected changes were driven by independent main effects. In kidney tissue, HBO was associated with lower potassium, magnesium, phosphorus, zinc, and lead levels, while PA was associated with lower iron and arsenic concentrations; both interventions were associated with lower sodium and mercury levels. In liver tissue, both HBO and PA were associated with reduced sodium levels, whereas HBO was associated with lower potassium and PA with higher magnesium concentrations. Several additional elements, including phosphorus, manganese, copper, cobalt, and selenium, demonstrated significant main effects, although *post hoc* analyses did not identify specific pairwise group differences. Taken together, these findings suggest that short-term HBO and acute PA induce distinct organ-specific adaptations in redox balance and elemental homeostasis, predominantly through independent rather than interactive mechanisms.

## Data Availability

The datasets generated and analyzed during this study are partly publicly available in the repository: https://radar.ibiss.bg.ac.rs/handle/123456789/8068. Additional row data supporting the conclusions of this article will be made available by the authors, without undue reservation.
